# Examining Cervical Cancer Preventive Behaviors for Latinx Transmasculine Individuals among Medical Students

**DOI:** 10.3390/ijerph18030851

**Published:** 2021-01-20

**Authors:** Alíxida Ramos-Pibernus, Paola Carminelli-Corretjer, Mario Bermonti-Pérez, Nelmit Tollinchi-Natali, Coral Jiménez-Ricaurte, David Mejías-Serrano, Julian Silva-Reteguis, Fabian Moreta-Ávila, Malynie Blanco, Lellanes Justiz, Marta Febo, Eliut Rivera-Segarra

**Affiliations:** 1 School of Behavioral and Brain Sciences, Ponce Health Sciences University, Ponce 00732, Puerto Rico; pcarminelli15@stu.psm.edu (P.C.-C.); mbermonti@psm.edu (M.B.-P.); ntollinchi15@stu.psm.edu (N.T.-N.); cjimenez18@stu.psm.edu (C.J.-R.); mblanco@psm.edu (M.B.); ljustiz@psm.edu (L.J.); mfebo@psm.edu (M.F.); elrivera@psm.edu (E.R.-S.); 2 Independent Community Researcher, Ponce 00732, Puerto Rico; davidmejiasserrano@gmail.com; 3 Independent Community Researcher, San Sebastián 00685, Puerto Rico; jmsilvareteguis@gmail.com; 4 Independent Community Researcher, Vallejo, CA 94590, USA; fabian.dmavila@gmail.com

**Keywords:** transmasculine, cervical cancer, Standardized Patient Simulations (SPS), medical students

## Abstract

Latinx transmasculine men (LTM) can be at a particularly high risk for cervical cancer as they lie at the intersection of two health disparity populations (gender and ethnic minorities). Previous research using self-report measures has documented how negative interactions with providers are a key barrier for cervical cancer screening among LTM. However, no research to date has examined, via direct observation, cervical cancer preventive behaviors in clinical interactions with LTM. Thus, the objective of this study was to examine cervical cancer preventive behaviors in clinical interactions between medical students and an LTM. The team implemented standardized patient simulations (simulations of clinical interactions with actors portraying the role of a patient), self-report measures, and observational techniques. A total of 37 medical students participated in the study. The results were mixed with some key behaviors neglected (i.e., asking if the patient preferred to collect the HPV test sample by himself), while others were enacted (i.e., checking family history of cervical cancer). Further research is needed to better understand behaviors in clinical interactions with LTM as well as how to improve them.

## 1. Introduction

Transmasculine individuals (TM; assigned to the female sex at birth but who live their lives as men or within the masculine spectrum) who have a cervix may be at a higher risk of cervical cancer [1,2,3]. Research has documented that TM patients are screened less and therefore, have lower odds of being up-to-date with cervical cancer screening compared to cisgender women (persons whose gender identity correspond to the assigned sex at birth) [4,5]. Some of the drivers for this disparity include stigma and discrimination in health care interactions, barriers to access cancer screening, lack of representation in the national cancer statistics, and lack of tailored cancer screening programs [6]. Latinxs are a particularly vulnerable group as they have the highest incidence of cervical cancer among all ethnic groups in the United States (US) [7]. Thus, Latinx TM (LTM) could be at an even higher cervical cancer risk as they lie at the intersection of two health disparity populations (gender and ethnic minorities).

Healthcare providers (i.e., physicians, advanced practitioners, nursing staff) are in a key position to foster cervical cancer prevention efforts [8,9,10,11]. However, research has documented that when interacting with healthcare providers, LTM often feel stigmatized and are provided with substandard levels of care [12,13]. These negative experiences can often lead LTM to avoid seeking healthcare altogether [13]. Furthermore, despite some provider’s intentions to provide high-quality healthcare to LTM, their lack of training and knowledge about LTM-specific healthcare needs, such as cervical cancer, might lead them to interact with patients in ways that can be perceived as negative [6,14,15,16,17]. For example, research has documented how offering a self-swab option for human papillomavirus (HPV) testing (as opposed to provider-collected) can be a patient-centered behavior that increases cervical cancer screening among this population [5,18]. Nevertheless, the team did not find any research effort to date assessing patient–provider clinical interactions with LTM via direct observation. This is an important gap, as interviews or self-report measures can only provide a limited understanding of cervical cancer prevention behaviors during patient–provider clinical interactions. Considering this, the objective of this study was to examine cervical cancer preventive behaviors among medical students during simulated clinical interactions with an LTM.

## 2. Materials and Methods

### 2.1. Design and Procedures

This study is part of a larger project examining barriers and facilitators for LTM cervical cancer screening. The research was approved by the Ponce Health Sciences University Institutional Review Board (1903007737). To address the purpose of this study, the team implemented Standardized Patient Simulations (SPS), self-reported measures, and observational techniques. SPS are simulations of clinical interactions with actors portraying the role of a patient, which are routinely used in medical schools to examine medical student’s competencies.

The total sample consisted of 37 third-year medical students, all of which were already scheduled to participate in their third-year SPS evaluations. Participants were recruited by availability and met the following criteria: (1) 21 years of age or older and (2) in the third year of medical school. The SPS program staff informed potential students about the study. Those interested were provided with an orientation and proceeded to complete the consent form and sociodemographic questionnaire before engaging in an SPS simulation with an LTM. The SPS case script, jointly developed by the research team (researchers, LTM, and SPS staff), presented an LTM with a chief complaint of pelvic pain and irregular bleeding. The actor had to (1) uniformly present the chief complaint and symptoms, (2) report a double mastectomy (if prompted), (3) disclose gender identity (if prompted), (4) report current testosterone use (if prompted), (5) report history of cervical cancer in the family (if prompted), and (6) report no previous history of HPV vaccination or screening (if prompted). These interactions lasted for approximately 20 min in which medical students had to (1) obtain the patient’s medical history, (2) discuss identified symptoms and risks, and (3) recommend treatment and testing. An LTM actor trained by the SPS staff in the study’s script performed all cases. This LTM SPS was interspersed among their other scheduled SPS cases. All participants were aware they would engage in an observed SPS with a LTM at some point during their SPS rotations. All SPS interactions were video recorded in order to be analyzed later using the behavioral measure described in the next section.

### 2.2. Measures

Sociodemographic Questionnaire—The team developed this self-report questionnaire to address participant’s demographic information such as age, gender, income, marital status, etc.

Cervical Cancer Preventive Behaviors Inventory (CCPBI)—This observational inventory was developed by the research team based on their previous research [19,20], establishing observational measures for SPS with the input of LTM participants and consultants. The CCPBI includes 38 nonverbal and verbal behaviors that can be enacted during a clinical interaction with an LTM. It assesses behaviors that are relevant to any interaction with a patient (general behaviors), and behaviors applicable to LTM (gender affirming behaviors and cervical cancer preventive behaviors). A trained observer assesses all behaviors using a three-point scale with the following values: manifested, unsure, and not manifested.

### 2.3. Data Analysis

Given the descriptive nature of this study, the team used one-way frequency tables to describe the sample and the general, gender-specific, and cancer-specific behaviors.

## 3. Results

### 3.1. Sample Characteristics

The total sample consisted of 37 students in their third year of medical school. Participants had a mean age of 36 years (SD = 2.0) and consisted mainly of single (67.6%), heterosexual (89.2%) cisgender females (54.1%) who lived in an urban area (97.3%). Participants identified themselves as Catholics (37.8%) and reported an annual income equal to or lower than $50,000. Participants had not received any type of training focused on transgender health issues (Table 1).

### 3.2. One-Way Frequency Analysis

The documented clinical skills in the SPS with LTM evidenced a concerning scenario. Findings suggest that while some general behaviors were manifested (i.e., answered the patient’s questions (100%); discussed family history of cervical cancer (94.3%); asked about current gender identity (74.3%)), others more specific to cervical cancer prevention among LTM were largely absent (i.e., exploring the patient’s reasons for avoiding visits to the doctor (77.1%), asking if the patient preferred to collect the sample by himself (85.7%); exploring potential discomforts the patients might have experienced in the waiting room (100%)). A more detailed description of these behaviors is presented in Table 2.

## 4. Discussion

In this study, the team aimed to examine medical students’ cervical cancer preventive behaviors when interacting with an LTM by using SPS and observational techniques. The findings suggest an alarming scenario in which medical students seem to lack important clinical skills for engaging in cervical cancer prevention efforts with LTM.

The main finding is that medical students in this study did not manifest key behaviors to address cervical cancer prevention when interacting with a LTM. Despite previous evidence emphasizing the importance of gender affirming behaviors in clinical interactions with LTM [13,20,21], some participants did not ask about the patient’s chosen name or pronoun, and used gendered language throughout the clinical interaction. Furthermore, none asked about potential concerns/discomfort while in the waiting room area. In addition, key cervical cancer preventive behaviors recommended by guidelines and recent research evidence [6,22,23,24,25,26] were not exhibited, such as asking about hysterectomy, checking for specific symptoms linked to cervical cancer, and asking if self-sample collection for screening was preferable. This could be related to the reported lack of training focused on transgender-related health issues. However, it could also point towards another interesting finding—that most trainings and guidelines focus on education through knowledge acquisition and attitude change, assuming that it will foster better behaviors in clinical interactions with LTM. Thus, these descriptive findings from actual behaviors in clinical interactions echo recent research arguing the need for surpassing the general Lesbian, Gay, Bisexual, and Transgender (LGBT) awareness and sensitivity training taught in medical schools by focusing instead on separate transgender-specific content, clinical skill building, and measured outcomes [14,27]. However, it is also important to highlight that these findings are mixed. For example, some participants did demonstrate gender-affirming behaviors (i.e., asking about current gender identity and avoiding making discouraging comments about hormone use). Similarly, some cervical cancer preventive behaviors were enacted by medical students, such as discussing family history of cervical cancer and referring to cancer screening tests.

Despite these findings, this study has several limitations to be noted. First, the team did not examine real patients, which does not allow for an examination of patient outcomes. Second, the small sample size and lack of control group limits the generalizability of the study findings. Third, participants were aware they would not encounter actual patients, which can prompt them to change their behaviors. Thus, the findings should be interpreted with caution. However, despite these limitations, this study addresses an important gap in the research literature, by focusing on actual observed behaviors rather than relying on self-report measures to better understand cervical cancer preventive behaviors for LTM and can serve as the basis for future research in the area.

## 5. Conclusions

Observed behaviors in clinical interactions between medical students and LTM were mixed, with some recommended behaviors being neglected and others enacted. Further research efforts addressing the limitations in this study are needed to better understand behaviors in clinical interactions with LTM as well as how to improve them.

## Figures and Tables

**Table 1:** Sample characteristics.

Variable	*N*	%
Assigned at birth sex		
Male	17	45.9
Female	20	54.1
Sexual orientation		
Heterosexual	33	89.2
Homosexual/Lesbian	2	5.4
Bisexual	2	5.4
Home area		
Urban	36	97.3
Rural	1	2.7
Marital status		
Single	25	67.6
Married	7	18.9
I live with my partner (not legally married)	5	13.5
Religious group		
Catholics	14	37.8
Protestants	6	16.2
None	13	35.1
Other	4	10.8
Annual income ((USD)		
Less than $50,000	19	51.4
From $50,001 to $60,000	2	5.4
From $60,001 to $70,000	4	10.8
From $70,001 to $80,000	1	2.7
From $80,001 to $90,000	1	2.7
From $90,001 to $100,000	3	8.1
More than $100,000	7	18.9
Training, seminar, or workshop related to transgender health issues		
Yes	6	16.7
No	30	83.3
Training on social/psychological aspects of transgender health		
Yes	6	16.7
No	30	83.3
Training on body modifications and transgender health		
Yes	2	5.6
No	34	94.4
Completed hours of training		
0	24	80
1	1	3.3
4	4	13.3
5	1	3.3

**Table 2:** Patient-provider clinical interaction behaviors.

	Not Manifested		Manifested	
	*N*	%	*N*	%
**General Behaviors**				
Answered the patient’s questions.	0	0	35	100
Used complex vocabulary to explain a condition or treatment to the patient.	32	91.4	3	8.6
Had adequate visual contact with the patient.	1	2.9	33	94.3
Explored psychosocial factors that might be linked to the patient’s situation (i.e., support group, transportation, access to care, etc.).	21	60	14	40
Addressed the patient’s concern regarding family conflict and/or explored how it might be linked to the patient’s current situation.	30	85.7	5	14.3
Showed a condescending attitude (childish style).	14	40	21	60
Showed a condescending attitude (dictatorial style).	26	74.3	5	14.3
Explored the patient’s reasons for avoiding visiting doctors.	27	77.1	8	22.9
Explained physical examination procedures (i.e., identify abnormalities, pain).	4	12.1	29	87.9
Asked if patient was comfortable with physical contact before proceeding to physical exam.	26	78.8	7	21.2
Recommended or prescribed pain medication.	33	94.3	2	5.7
**Gender Affirming Behaviors**				
Explored the name and pronouns the patient prefers.	21	60	14	40
Asked about current gender identity.	9	25.7	26	74.3
Asked questions related to patient’s at-birth genitalia.	14	40	20	57.1
Confused or assumed birth genitals.	26	74.3	5	14.3
Asked if patient is sexually active.	3	8.6	32	91.4
Explored patient’s sexual history (penetrative or receptive).	29	82.9	5	14.3
Explored safe sex practices (i.e., condom use).	10	28.6	25	71.4
Discussed gender-affirming surgeries (i.e., mastectomy, reassignment surgery, hysterectomy).	2	5.7	33	94.3
Explored if the patient is currently using hormones.			35	100
Explored if hormone treatment is medically supervised.	19	54.3	16	45.7
Made discouraging comments about hormone use.	35	100		
Explored potential concerns and/or discomfort that patient might have experienced in the waiting room.	35	100		
Used gendered language (i.e., vagina, menstruation, uterus, ovaries, cervix).	19	54.3	16	45.7
Explored previous experiences with doctors (i.e., negative patient/providers interactions).	30	85.7	4	11.4
**Cervical Cancer Preventive Behaviors**				
Explored previous gynecological checkups.	25	71.4	10	28.6
Discussed family history of cervical cancer.	2	5.7	33	94.3
Asked about hysterectomy.	17	54.8	13	41.9
Asked if patient had HPV vaccination.	19	54.3	16	45.7
Explored specific symptoms linked to cervical cancer (i.e., pelvic pain, irregular bleeding).	18	51.4	17	48.6
Asked about previous cancer screening.	8	22.9	27	77.1
Explained cancer screening procedures.	24	68.6	11	31.4
Explained HPV and its link to cancer.	20	57.1	14	40
Asked if the patient preferred to collect the sample by himself (i.e., self-swab).	30	85.7	5	14.3
Offered alternatives if patient expressed discomfort during clinical encounter and/or physical exam (i.e., keep shirt, come back another day).	5	14.3	30	85.7
Referred a cancer screening test.	1	2.9	34	97.1
Managed patient’s concerns regarding gynecologist referral.	13	37.1	22	62.9
Recommended HPV vaccine.	21	60	14	40
Offered recommendations to address chief complaint (i.e., STD test, pain management strategies).	1	2.9	34	97.1

Note: *N* = 35 due to some missing behavioral data.

## Data Availability

Data is contained within the article.
